# Connexins, Innexins, and Pannexins: From Biology to Clinical Targets

**DOI:** 10.3390/biom11020155

**Published:** 2021-01-25

**Authors:** Trond Aasen

**Affiliations:** 1 Patologia Molecular Translacional, Vall d’Hebron Institut de Recerca (VHIR), Vall d’Hebron Hospital Universitari, Vall d’Hebron Barcelona Hospital Campus, Passeig Vall d’Hebron 119-129, 08035 Barcelona, Spain; trond.aasen@vhir.org; 2 CIBER de Cáncer (CIBERONC), Instituto de Salud Carlos III, Avenida de Monforte de Lemos 3-5, 28029 Madrid, Spain; 3 Universitat Autònoma de Barcelona, 08193 Bellaterra, Spain

In 1964, Loewenstein and Kanno [1,2] used *Drosophila flavorepleta* larvae to demonstrate direct intercellular communication involving the transmission of ions and tracer dyes between salivary gland epithelial cells. This phenomenon—later shown to be mediated by specific channel proteins and to occur in nearly all cell types and across the animal kingdom—is critical for growth and development, tissue homeostasis, and specific organ functions. In this Special Issue (SI), we cover the role of the connexin, innexin, and pannexin channel proteins in this setting, shedding light on various aspects of a multidisciplinary field that is rapidly evolving with the discovery of new properties, biological functions, and phenotypic associations. This new insight is leading to novel therapeutic opportunities to tackle some of the associated diseases, which include deafness, blindness, skin disease, cancer, cardiovascular disease, and a wide variety of neurological disorders and complex syndromic diseases.

In chordates, including vertebrates such as humans, gap junctions are formed by a large family of four-transmembrane connexin proteins that assemble into hexameric channel pores, named connexons, that dock with connexons in an adjacent cell [3]. This allows for direct intercellular communication that facilitates the exchange of small molecules between cells, such as ions, metabolites, second messengers, small peptides, and even microRNAs. In addition, under certain pathophysiological conditions, connexons can act as hemichannels to allow for the direct exchange of small molecules between the cell cytosol and the extracellular milieu. Furthermore, a myriad of non-canonical connexin functions have been identified, with roles for connexins as parts of signaling hubs, as components of mitochondria, and as regulators of extracellular vesicle biology. The direct translation of truncated non-junctional connexin forms has added another layer of complexity to this puzzle. Non-chordates, on the other hand, such as flatworms and the aforementioned fruit fly *Drosophila*, use the innexin protein family to form octameric structures that assemble into hexadecameric intercellular channels that serve a similar purpose [4]. Based on their homology to the innexin gap junction genes, the pannexin family (Panx1–3) was discovered in vertebrates in 2000 [5]. Initially proposed to form cell–cell gap junctions, this protein family was subsequently found to predominantly form single-membrane heptameric channels that connect the intracellular and extracellular space [6].

The families of these transmembrane proteins—the connexins, innexins, and pannexins—have been extensively studied since their discovery, and form a vibrant and diverse field with its own biennial international congress running since 1974, the next of which is due to take place in A Coruña (Spain) in July 2022 (https://igjconference.org/). The recent intense and broad activity in this area of research is showcased in this SI, which comprises 15 original research articles and six review articles.

The interest in this field is partly due to the close association between these proteins and many human diseases. At least 28 human genetic diseases are linked to mutations in 10 out of the 21 members of the human connexin gene family, with mutations in the connexins Cx26 and Cx43 causing at least 14 disorders [7]. Notably, mutations in Cx26, encoded by the *GJB2* gene, are the most common cause of inherited prelingual deafness worldwide. Apart from clinical mutational screening, considerable effort is being expended in understanding the pathogenicity of different mutations. In this SI, Maslova et al. [8] report how they used CRISPR/Cas9 *GJB2* knockout cells for functional analysis of the rare *GJB2* mutation c.516 G > C (p.Trp172Cys) to show that this W172C mutation—localized to the second extracellular loop of Cx26—impairs Cx26 trafficking to the membrane and thus hemichannel permeability.

Undoubtedly, the best-studied connexin member is Cx43, a phenomenon also reflected in this SI, with 11 articles focusing on this entity. Mutations in Cx43 can cause a variety of symptoms and diseases, including complex syndromic diseases affecting multiple organs [7], most notably oculodentodigital dysplasia (ODDD). Shao et al. [9] demonstrate, through induced pluripotent stem cell approaches (including cells obtained from an ODDD patient), that Cx43 is dispensable for the adipogenic differentiation of early-stage human mesenchymal stem cells but protects against cell senescence. On the other hand, Au et al. [10], from the same group, show that CRISPR/Cas9 ablation of Cx43 reduces overall gap junction coupling in monolayer cultures of rat epidermal keratinocytes (REKs) and dysregulates the differentiation of REKs grown in organotypic cultures. These two articles nicely highlight the many distinct tissue-specific functions of Cx43. They also show that Cx31 displays a different life expectancy compared with Cx43 in REKs and forms gap junctions that are mobile within the membrane—undergoing both fusion and fission events—further indicating the existence of different connexin isoform-specific properties within the same tissue [10].

Unlike Cx31, the turnover of Cx43 has been extensively studied. In this SI, Solan and Lampe [11] highlight the importance of the C-terminal phosphorylation of Cx43 in this setting, showing that Src activation promotes the formation of internalized Cx43 gap junction structures, known as connexisomes, through ERK-mediated phosphorylation of S279/282. Using proteasomal and lysosomal inhibitors, they present data and a model in which phosphorylation regulates multiple modes of Cx43 gap junction disassembly.

In addition to the large Cx43 “kinome”, the Cx43 interactome is also extensive. In this regard, Zheng et al. [12] describe three ODDD mutations occurring in the calmodulin-binding motif of the Cx43 cytoplasmic loop that causes either cytoplasmic retention or non-functional channels. They reveal that calmodulin interacts with the part of the Cx43 C-terminal tail region in which tyrosine residues are phosphorylated by Src and Pyk2.

An important interplay also occurs between Cx43 and the actin cytoskeleton. Strauss and Gourdie [13] review this topic in-depth, with particular focus on how both canonical and non-canonical Cx43 functions can regulate the tissue barrier and how this newfound insight and the development of targeted therapies such as Cx43 mimetic peptides can be used to dissect and improve the outcome of barrier-associated diseases.

As illustrated above with regard to the regulation of Cx43 and multi-organ diseases such as ODDD, the mechanism by which connexins cause disease is complex and tissue-dependent. In the eye, mutations in Cx46 or Cx50 can cause cataracts. Jara et al. [14] hypothesized that mutations in these connexins would impair lens glutathione metabolism and thereby facilitate oxidative damage. Although they showed that both Cx50D47A and Cx46fs380 mutant mouse models have oxidizing lens environments, oxidative modification is greater in Cx50D47A mice than in Cx46fs380 mice. They conclude that the loss of glutathione permeation through lens connexin channels and oxidative damage is likely not a decisive early event in cataract formation in these mice, and that the involvement of cell–cell permeabilization through lens connexins clearly remains a critical and potential therapeutic angle in disease progression.

One of the most multifactorial diseases is cancer. Connexins regulate all of the hallmarks of cancer, but in a complex manner, bestowing both pro- and anti-tumorigenic properties through the action of gap junctions, hemichannels, and non-junctional properties, as reviewed in this SI by Mulkearns-Hubert et al. [15]. While summarizing all of these hallmarks, the authors reiterate the need for progress in connexin-modulating approaches to help in elucidating functions and developing specific clinical therapies. The “Jekyll and Hyde” role for connexins in cancer is highlighted [15] and is an obvious challenge. Most cancer-related studies concern Cx43, for which this dichotomous role is prominent. In this SI, Talbot et al. [16] detail the complex functions of this protein in bone tumors, with the overall evidence pointing toward an antagonistic role as a tumor suppressor in the development of primary bone tumors and as a tumor promoter in relation to secondary bone tumors. However, focus is now being given to the dissection of the function of other connexin family members, including their non-canonical features. This is illustrated by Acuña et al. [17], who report that Cx46 in extracellular vesicles can promote malignant features in breast cancer cells.

The role of connexins also features prominently in the pathophysiology of heart disease, the leading cause of death worldwide. The canonical and non-canonical roles of Cx43 in cardioprotection are reviewed here by Rusiecka et al. [18]. Cx43 is a predominant channel protein in cardiomyocytes, but is also present in mitochondria and, as reviewed, our understanding of Cx43 in heart disease, particularly in ischemia, has also resulted in the development of Cx43-mimetic peptides that successfully diminish cardiac injury in preclinical studies [18]. In this respect, Valls-Lacalle et al. [19] used their established Cx43 mouse model to assess changes in scar size and left ventricular remodeling upon transient myocardial ischemia followed by reperfusion. They concluded that Cx43 deficiency has a protective effect on scar formation after transient coronary occlusion, an effect associated with reduced left ventricular remodeling and attenuated elevation of pro-transforming growth factor beta 1 expression. Analysis of Cx43 remodeling in heart disease is a significant area of research with potential clinical utility. In this respect, Oliver-Gelabert et al. [20] describe an open source software algorithm—Myocyte Automatic Retrieval and Tissue Analyzer (MARTA)—that automatically quantifies Cx43 lateralization in cardiomyocytes. Cx45 is also expressed in the heart, but additionally seems to play a prominent role in the lymphatic system, for example, by mediating lymphatic muscle cell coupling. Castorena-Gonzalez et al. [21] used their Cx45-deficient mouse model to show how this connexin isoform regulates lymphatic pacemaking upon elevated downstream pressure. The more recently described pannexin channel proteins also seem to play a role in the vasculature, with multiple studies in mice pointing to a pathological role for Panx1. With regard to this, Molica et al. [22] describe a single nucleotide polymorphism in Panx1 (Panx1-400A > C) that predisposes non-obese humans to body mass index-dependent endothelial dysfunction.

Our deeper understanding of the disease-causing mechanisms of these channel proteins has also significantly spurred the development of therapeutic approaches. Freitas-Andrade et al. [23] describe an antiarrhythmic dipeptide (danegaptide) that seems to cross the blood–brain barrier and enhance astrocytic Cx43 coupling. Administration of the peptide in a novel mouse brain ischemia/reperfusion model significantly decreased infarct volume, providing evidence for therapeutic potential in ischemia/reperfusion stroke. Astrocytic Cx43 is also a key candidate for the treatment of epilepsy, and this specific area of research is extensively reviewed by Walrave et al. [24]. Indeed, numerous neurological disorders are linked to gap junctions. In a highly comprehensive review, Mesnil et al. [25] summarize the role of gap junctions in the parenchyma and how various pollutants may affect connexins in this setting, and finally describe the major brain disorders (neurodevelopmental, neurobehavioral, neurodegenerative, cancer) linked to both connexin dysfunction and pollutants.

As mentioned above, the very first breakthrough in the field was obtained through more simplistic animal models, such as *Drosophila*, which do not even contain any connexin genes. The field continues to benefit from such non-vertebrates and, in this SI, Starich and Greenstein [26] describe a set of innexin 8 (INX-8) mutants in the nematode *Caenorhabditis elegans* to gain insight into the role of INX-8 in germline proliferation and gametogenesis. They provide a model to further understand channel function and elucidate regulatory biomolecules that cross soma–germline gap junctions.

Although the chordate pannexin gene/protein family was identified based on its similarities to innexins, these single-membrane channel proteins have distinct properties and functions, many of which remain to be elucidated. Timonina et al. [27] used site-specific mutagenesis, dye uptake assays, and interaction tests to identify two conserved aromatic residues, Trp123 and Tyr205, in transmembrane domains 2 and 3 of the zebrafish Panx1 protein. The authors suggest that these residues govern channel assembly and provide mechanistic insight into Panx1 oligomerization, glycosylation, and trafficking.

As a model for a human connexin-deficient cell line, HeLa cells are probably the most extensively used. However, weak gap junctional activity has been noted in this cell line and, based on electrophysiological recordings, this activity has been suggested to be mediated by Cx45. Choi et al. [28] use the iodide-yellow fluorescent protein assay and CRISPR technology to provide direct proof that this is the case and suggest that HeLa Cx45 knockout cells would be a more suitable model for gap junction-deficient cells.

To conclude, progress is being made on multiple levels in this field—as highlighted in this SI—providing valuable biological insight into connexin, innexin, and pannexin function. As we decipher the vocabulary, we can now start to translate the language of intercellular communication and its role in pathophysiology, allowing us to answer key clinical questions and provide novel therapeutic solutions.
